# Two new cellulolytic fungal species isolated from a 19^th^-century art collection

**DOI:** 10.1038/s41598-018-24934-7

**Published:** 2018-05-10

**Authors:** Carolina Coronado-Ruiz, Roberto Avendaño, Efraín Escudero-Leyva, Geraldine Conejo-Barboza, Priscila Chaverri, Max Chavarría

**Affiliations:** 1 Centro Nacional de Innovaciones Biotecnológicas (CENIBiot), CeNAT-CONARE, 1174-1200 San Jose, Costa Rica; 2 Centro de Investigaciones en Productos Naturales (CIPRONA), Universidad de Costa Rica, 11501-2060 San Jose, Costa Rica; 3 Escuela de Biología, Universidad de Costa Rica, 11501-2060 San Jose, Costa Rica; 4 Escuela de Química, Universidad de Costa Rica, 11501-2060 San Jose, Costa Rica; 5 Instituto de Investigaciones en Arte (II Arte), 11501-2060 San Jose, Costa Rica; 6 Department of Plant Science and Landscape Architecture, University of Maryland, College Park, MD 20742 Maryland, USA

## Abstract

The archive of the Universidad de Costa Rica maintains a nineteenth-century French collection of drawings and lithographs in which the biodeterioration by fungi is rampant. Because of nutritional conditions in which these fungi grew, we suspected that they possessed an ability to degrade cellulose. In this work our goal was to isolate and identify the fungal species responsible for the biodegradation of a nineteenth-century art collection and determine their cellulolytic activity. Fungi were isolated using potato-dextrose-agar (PDA) and water-agar with carboxymethyl cellulose (CMC). The identification of the fungi was assessed through DNA sequencing (nrDNA ITS and α-actin regions) complemented with morphological analyses. Assays for cellulolytic activity were conducted with Gram’s iodine as dye. Nineteen isolates were obtained, of which seventeen were identified through DNA sequencing to species level, belonging mainly to genera *Arthrinium, Aspergillus, Chaetomium, Cladosporium, Colletotrichum, Penicillium* and *Trichoderma*. For two samples that could not be identified through their ITS and α-actin sequences, a morphological analysis was conducted; they were identified as new species, named *Periconia epilithographicola* sp. nov. and *Coniochaeta cipronana* sp. nov. Qualitative tests showed that the fungal collection presents important cellulolytic activity.

## Introduction

Variations in the composition and appearance of a material as a consequence of the action of microorganisms is known as biodeterioration^1^. This phenomenon becomes evident with the presence of reddish-brown or yellowish-brown patches, microfungal structures and textural changes, which are commonly found in ancient documents^2^. These conditions apply to a nineteenth-century French collection of drawings and lithographs by Bernard Romain Julien (1802–1871) that is held in the archive of the School of Plastic Arts of Universidad de Costa Rica. The damage due to the microbial proliferation in these works of art is related to the storage conditions, especially to the damp and warm environments^3^. To design an effective and specific treatment according to the species growing in the laminae led to the isolation and identification of the fungal species responsible for the foxing of the lithographs.

Previous investigations of the microbiota in antique documents reported the presence of fungi that belong mainly to genera *Alternaria, Aspergillus*, *Chaetomium*, *Cladosporium*, *Penicillium*, and *Trichoderma*^1,2,4–7^. For instance, El Bergadi *et al*. (2013) isolated, identified and characterized the microbiota of manuscripts from an ancient collection of the Medina of Fez and found *Aspergillus niger*, *Aspergillus oryzae, Mucor racemus*, and *Penicillium chrysogenum*, as the most frequent species from a total of 31 fungal isolates^8^.

Because of nutritional limitations in which these fungi grew and where cellulose of the laminae was the only source of carbon, the species responsible for the biodeterioration were believed to possess cellulolytic activity^8,9^. This cellulolytic activity is of interest for multiple biotechnological processes, such as treatment of agroindustrial residues^10,11^ or production of cellulases^12^. This condition was first deduced and published in 1903 by van Iterson in “La décomposition del la cellulose pas les microrganismes”^7^. An investigation of the microbial diversity in a nineteenth-century Islamic and Koranic book led to the discovery of nine fungal species with the ability to degrade carboxymethylcellulose (CMC), including *Aspergillus niger*, *A. oryzae* and *Hypocrea lixii*^8^. Michaelsen *et al*. (2009) and Pinzari *et al*. (2006) described *Aspergillus versicolor*, *A. nidulans*, *A. terreus* and *Chaetomium globosum* as agents in the microbiological damage of old documents^5,6^.

Given the interest in the developing methods for protecting and preserving ancient documents from microbial degraders^13^ and the importance of obtaining microorganisms or enzymes with the capacity to degrade lignocellulosic wastes^14^, the aim of the present work was to isolate and identify the fungal species responsible for the biodegradation of a nineteenth-century art collection and to determine their cellulolytic activity. We found 19 fungal isolates belonging mainly to genera *Arthrinium*, *Aspergillus, Chaetomium, Cladosporium, Colletotrichum, Penicillium and Trichoderma*. Two samples not identified through their DNA sequences were identified through morphological analysis as new fungal species, namely *Periconia epilithographicola* sp. nov. and *Coniochaeta cipronana* sp. nov. Qualitative tests showed that the fungus collection presents important cellulolytic activity.

## Methods

### Sampling and isolation of cellulolytic fungi

A total of 13 laminae from a nineteenth-century French collection of lithographs belonging to Universidad de Costa Rica with signs of biodeterioration were sampled in areas of critical damage (colored or discolored areas, microfungal structures or other observable textural changes in the paper) with sterile cotton swabs, which were subsequently submerged in Phosphate Buffered Saline (PBS, 100 µL). Samples (50 µL) were cultured onto potato dextrose agar (PDA; Difco Potato Dextrose agar, BD company, France), and onto water agar with carboxymethyl cellulose (CMC, 1%, Sigma-Aldrich) with kanamycin (km, 50 µg/mL, Sigma-Aldrich). Morphologically distinct colonies were isolated and purified onto plates with the same culture media^2,15–17^.

### Molecular identification

To identify the various fungal isolates, DNA extraction was performed using the method described by Lodhi *et al*. (1994) with modifications^18^. First, two disks (diameter 0.8 cm) from each fungal colony were introduced into Eppendorf tubes (1.5 mL). An extraction buffer (750 µL) was added, followed by the vortex of the sample and an incubation period (30 min at 67 °C). DNA was then precipitated with the addition of a CHCl_3_:octanol mixture (24:1, 750 µL), separation of the supernatant, and addition of isopropanol (600 µL, Sigma-Aldrich) and ethanol (500 µL, 70% v/v, Sigma-Aldrich). The DNA was eventually resuspended in AE buffer (50 µL, Qiagen) containing RNAse (1 µL, Fermentas). PCR reactions were performed to amplify the ITS (ITS 4 and ITS 5) and actin (Act-512F and Act-783R) regions using a reaction mix (PCR Master Mix, 10 µL, 2X, Thermo Scientific), water (7 µL), primers (0.5 µL, 10 μM) and DNA (2 µL, 50 ng/μL)^19,20^. All PCR reactions were performed in a thermocycler (Applied Biosystems 9902, Norwalk, USA) according to conditions described by Carbone & Kohn (1999) and White *et al*. (1990) for actin and ITS primers, respectively^19,20^.

The amplified products were purified with a clean-up kit (EXO-AP, Thermo Scientific, USA) and sequenced with a genetic analyzer (ABI 3130*xl*) and a reaction kit (Big Dye v.3 Terminator Cycle Sequencing Ready Reaction Kit, Applied Biosystems, USA), using ITS and actin primers (1 μM). Sequences were analyzed with software (MEGA 7), and were run through a Standard Nucleotide BLAST (Genbank, NCBI nucleotide database) to assess the similarity with reported sequences of fungal species. The BLAST searches were run excluding uncultured/environmental samples in the database. To corroborate the results, the BLAST search was repeated limiting the search to sequences only from type material. All sequences have been deposited in the GenBank database under the accession numbers that appear in Supplementary Table S1.

### Morphological identification

Two species that did not have a close match to anything in Genbank, were examined in more detail to determine their morphological characteristics. Morphological analyses followed recommendations and techniques described by Ellis (1971) for hyphomycetous fungi and common methods in mycology^21,22^. Fungal isolates were cultured in CMD (BBL Corn Meal Dextrose agar, BD Company, France) and PDA (Difco Potato Dextrose agar, BD company, France) for 7 to 10 days near 25 °C. An optical microscope (Olympus BX-40, Japan) was used with an attached camera (18 megapixels, OMAX, Korea); software (ToupView, ToupTek Photonics, China) was used to measure structures.

### Screening of cellulolytic activity

Cellulase-producing microorganisms were screened on agar plates enriched with only CMC as a source of carbon, with Gram’s iodine as indicator (Prelab)^23–26^. This qualitative determination is based on the interaction of iodine with cellulose and its components in its degraded form, such that the integral biopolymer holds Gram’s iodine dye; whereas areas with cellulose hydrolyzed by enzymes result in clear zones or the appearance of a pale halo^15,27^. The halo was measured for the subsequent calculation of the enzymatic index (EI), a semi-quantitative estimate of the enzyme activities, according to this formula^15^.

1 $${EI}=\frac{Diameter\,of\,hydrolysis\,zone}{Diameter\,of\,colony}$$

For this purpose, fungal discs (diameter 0.8 cm) were grown in a solid medium composed of water agar (1.6%), CMC (1%) and kanamycin (km, 50 µg/mL). After cultures were incubated (7 days, 30 °C), plates were flooded with Gram’s iodine stain (10 mL, 10 min) and washed with water to enable the observation, photographing and measurement of the clear zone around the fungal growth^23–26^. Software (ImageJ, version 1.51j8) was used to measure the diameters^28^. The experiment was repeated twice (on separate days) with duplicates of each isolate. *Pleurotus ostreatus* served as a positive control^29^.

## Results and Discussion

### Isolation and identification of fungi isolated from drawings and lithographs

**Figure 1:**
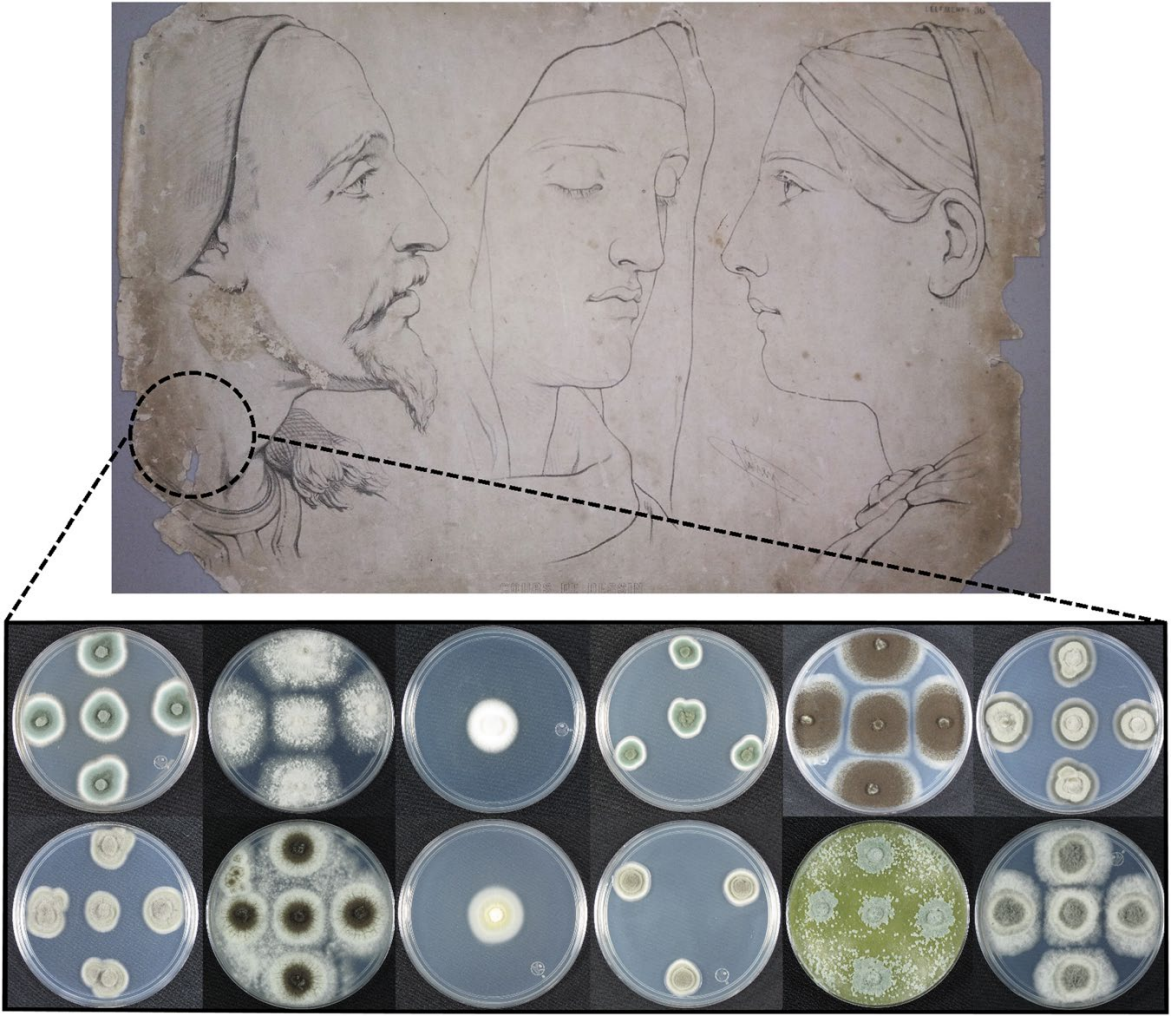
Fungal diversity in ancient lithographs. Several fungi were isolated from stained and degraded areas from nineteenth-century drawing laminae. On the top row from left to right are isolates #5, #10, #19, #9, #8, and #15. On the bottom row from left to right are isolates #16, #22, #21, #11, #23 and #26. Samples in the image were grown in PDA during 6 days. We thank Dr. Salomón Chaves (Instituto de Investigaciones en Arte) for authorizing the use of images from the collection of drawings by Bernard Romain Julien in this manuscript.

Through the screening of the lithographs, the total count of fungi isolated was 19, of which eight grew directly in water agar with CMC-km and eleven were first isolated from PDA and then recultivated in water agar with CMC as the sole source of carbon. The proliferation of fungi in the latter culture medium is in accordance with the environment in which they were isolated (limited sources of carbon, with cellulose as sole nutrient). Laminae #5 was the most contaminated, with ten isolations; followed by laminae #7 and #10, with 3 isolations each (see Supplementary Figure S1). The fungal isolates showed diverse forms, sizes, elevations, borders, surfaces, opacity, color and growth rates, as shown in Fig. 1.

BLAST searches in GenBank database resulted in the classification of nineteen isolates into fifteen species and nine genera (Table 1). These nineteen isolates had at least a 98% similarity with known species. The most prevalent genus was *Cladosporium*. Of the nine identified genera, *Aspergillus*, *Chaetomium, Cladosporium*, *Penicillium*, and *Trichoderma* are reported as common microbiota in ancient works of art^1,2,17,30,31^. The actin region was sequenced to confirm the results obtained with the ITS region, and to classify to species level some samples that could not be done with ITS. For all cases in which both ITS and actin sequences were obtained, the fungi were classified within the same species, except isolate #9 in which the actin region denied conclusive results obtained with ITS.

**Table 1:** Molecular identification of isolated fungi using ITS and actin regions.

Isolate#	Identification	ITS and closest accesion number			Actin and closest accesion number		
		Accession	Identity	Coverage	Accession	Identity	Coverage
4	*Cladosporium sphaerospermum*	KP701988.1	100%	100%	EU570272.1	98%	99%
5	*Penicillium chrysogenum* ^*a*^	KC009774.1	100%	100%	AM920435.1	97%	100%
6	*Penicillium westlingii* ^*b*^	JN617668.1	100%	100%	AM920435.1	83%	55%
7	*Cladosporium tenuissimum* ^*c*^	KP701937.1* KJ596320.1*	100%	100%	LN834582.1	100%	99%
8	*Aspergillus niger*	KJ365316.1	100%	100%	AM270331.1	99%	99%
9	*Cladosporium* sp.	KP701937.1*KJ596320.1*	100%	100%	—	—	—
10	*Arthrinium arundinis* ^*b*^	KF144889.1	100%	100%	AY951865.1	76%	76%
11	*Cladosporium angustisporum* ^*c*^	MG250413.1*MG199960.1* KP701978.1* KP701964.1* KP701938.1* KP701935.1* KP701930.1* KP701908.1*	100%	100%	LN834540.1	100%	100%
12	*Aspergillus versicolor*	NR_131277.1	100%	95%	—	—	—
13	*Chaetomium* cf. *subglobosum*^*b*^	NR_144826.1	96%	99%	KF545191.1	99%	100%
15	*Cladosporium angustisporum* ^*c*^	MG250413.1*MG199960.1* KP701978.1* KP701964.1* KP701938.1* KP701935.1* KP701930.1* KP701908.1*	100%	100%	LN834540.1	100%	100%
16	*Cladosporium cladosporioides* ^*c*^	MG250413.1*MG199960.1* KP701978.1* KP701964.1* KP701938.1* KP701935.1* KP701930.1* KP701908.1*	100%	100%	KT600582.1	99%	96%
17	*Chaetomium* cf. *subglobosum*^*b*^	NR_144826.1	100%	99%	KF545191.1	99%	100%
19	*Periconia* sp.^*d*^	HQ608027.1	99%	100%	KP184118.1	83%	95%
20	*Chaetomium cf. subglobosum* ^*b*^	NR_144826.1	99%	99%	KF545191.1	99%	100%
21	*Coniochaeta* sp.^*d*^	KX869958.1	99%	100%	AY579255.1	71%	100%
22	*Aspergillus niger*	KJ365316.1	100%	100%	AM270331.1	99%	99%
23	*Trichoderma* cf*. Longibrachiatum*^*b*^	KT336509.1	100%	100%	JQ238613.1	98%	99%
26	*Colletotrichum kahawae* ^*c*^	NR_144787.1	100%	98%	JX009431.1	99%	100%

^a^Isolates had homology with two fungi of different species with the ITS region analysis, but through sequencing of the actin region it was possible to confirm the identification.

^b^No register in the NCBI GenBank database for actin sequencing regions.

^c^ITS region sequencing allowed to identify only the isolates at genus level; the actin region enabled an identification at specie level.

^d^No register in the NCBI GenBank database for either ITS or actin sequencing regions.

*Accessions with same similarity.

Two isolates were only identified to genus or class levels using both ITS and actin regions. Specifically, isolate #19 was classified within the genus *Periconia*, and isolate #21 was classified within the class Sordariomycetes, both in the phylum Ascomycota. Since these two isolates did not have a close match to any sequence in the Genbank, traditional morphological analyses and descriptions (e.g. microscopy and use of taxonomic literature) were done to elucidate the identity of these isolates.

### Description of two new fungal species

#### Periconia epilithographicola C

**Figure 2:**
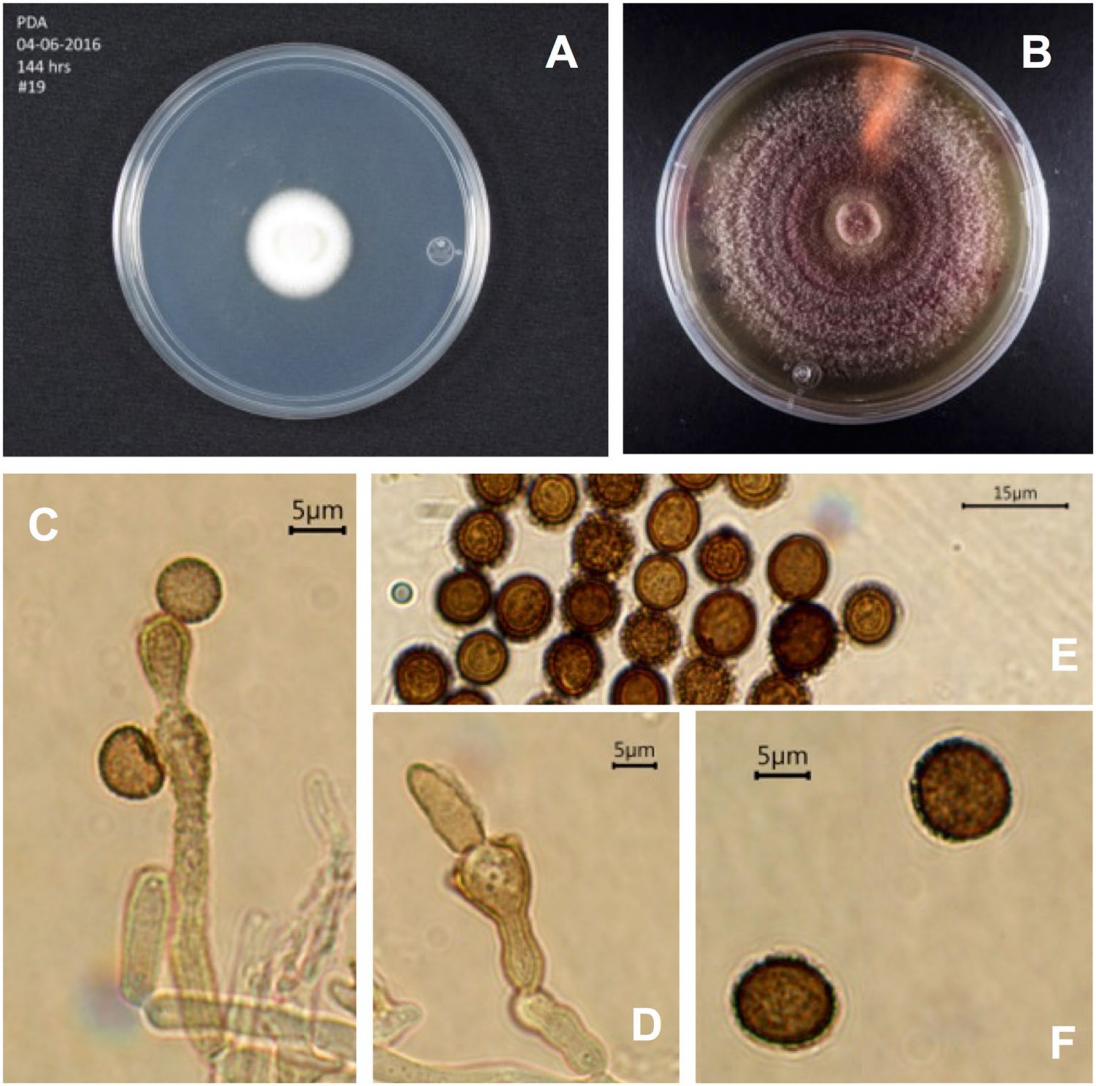
*Periconia epigraphicola*. (**A**) PDA Culture ca 6 days. (**B**), PDA Culture ca 20 days. (**C**) Conidiogenous cell forming conidia. (**D**) Conidiogenous cell. (**E**) Catenated spinulose conidia. (**F**) Spinulose conidia.

Coronado-Ruiz, R. Avendaño, E. Escudero-Leyva, G. Conejo-Barboza, P. Chaverri & M. Chavarría **sp**. **nov**. Fig. 2. **Mycobank**: MB825093 **GenBank**: MF422162 (ITS) & MF422179 (actin). **Etymology**: *epilithographicola*, because it was found growing over art lithographs. **Holotype:** Costa Rica, San José, San Pedro de Montes de Oca, Universidad de Costa Rica; on art lithographs; May 19th, 2016; collected by Avendaño R.; extype culture CBS 144017, a permanently preserved, metabolically inactive culture (=#19). **Diagnosis**: *Periconia* species producing a pinkish to reddish pigment. Straight conidiophores; globose, echinulated, golden-brown conidia. **Colonies**: At 25 °C after three weeks, on CMD, attaining 25 mm diam., colony white, cottony. On PDA, attaining 60 mm diam., colony effuse, pinkish (similar to OAC486), with creeping hyphae; conidiophores visible, forming small agglutinated black sticky drop-like structures. **Conidiophores**: macronematous, with creeping hyphae forming stipes 251.6–270 · 3.6–6.1 µm, straight, branched singly near the base, seven or more septate, grayish to black. **Conidiogenous cells:** holoblastic, (5.1−) 7 · 10 (−11.5) µm (n = 15), sub-globose to ellipsoid, finely roughened, yellowish to brown (slightly more brilliant than OAC757). **Conidia:** globose, (7.8−) 9.2 (−10.7) µm diam. (n = 30), golden to brown (similar to OAC705), echinulated, catenated, sometimes forming long chains. **Habitat:** Growing on aged lithographs of Instituto de Investigaciones en Arte (Universidad de Costa Rica). **Notes:** Several *Periconia* species share similar characteristics of the conidiophore, differing mainly in the conidia size. *Periconia pseudobyssoides* conidia are larger, (12−) 15–17 (−20) µm diam. and brown-reddish^32^; *P. byssoides* conidia are 10–15 µm in diam^21^.; *P. saraswatiurensis* conidia are 9–12 µm diam., also secreting dark green to purple pigments in culture^21^. The only species with a similar conidial size is *P. jabalpurensis* but it lacks septa in the conidiophores; *P. macrospinosa* shows conidia of up to 35 µm diam. with long spines (<2 µm)^21^, which does not fit *Periconia epilithographicola*.

#### Coniochaeta cipronana C

**Figure 3:**
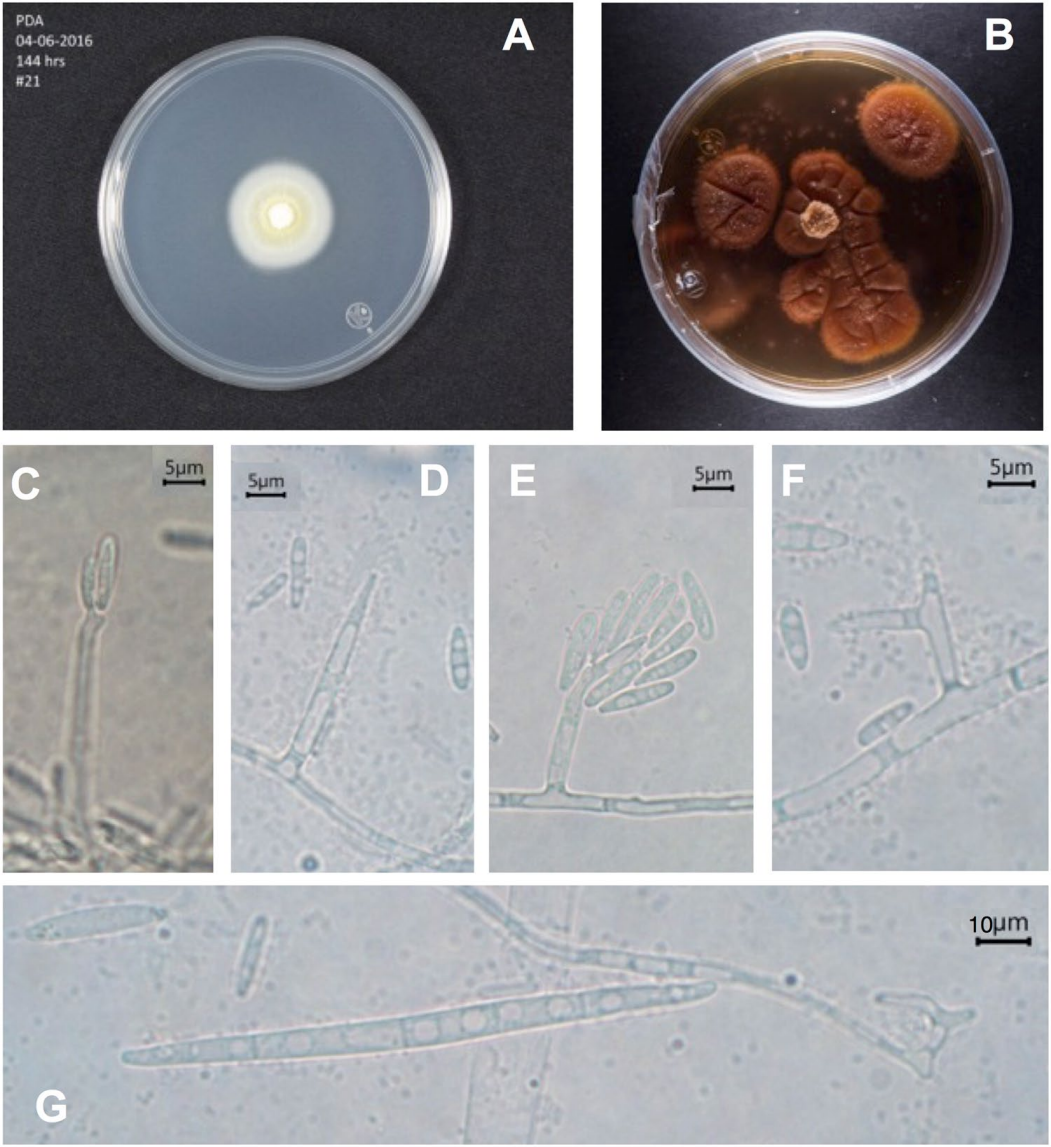
*Coniochaeta cipronana*. (**A**) PDA Culture ca 6 days. (**B**) PDA Culture ca 20 days. (**C**–**E**) Conidiogenous cell with small conidia. (**F**) Dicotomic conidiophore. (**G**) Macroconidia 5–7 septate.

Coronado-Ruiz, R. Avendaño, E. Escudero-Leyva, G. Conejo-Barboza, P. Chaverri & M. Chavarría **sp. nov**. Fig. 3. **Mycobank**: MB825094 **GenBank**: MF422164 (ITS) & MF422181 (actin). **Etymology**: as a reference to Centro de Investigaciones en Productos Naturales (CIPRONA, Universidad de Costa Rica) for the impact and transcendence of the research in the field of natural products over 38 years. **Holotype**: Costa Rica, San José, San Pedro de Montes de Oca, Universidad de Costa Rica; from art lithograph; May 19th, 2016; collected by Avendaño R.; extype culture CBS 144016, a permanently preserved, metabolically inactive culture (=#21). **Diagnosis:** *Nodulisporium*-like conidiophore, with macro- and microconidia, hyaline, macroconidia 5–7-septate slightly curved, fusiform, microconidia cylindrical 1–2-septate. **Colonies:** At 25 °C after 3 weeks on CMD, reaching 20 mm diam., hyaline to white. On PDA attaining 25 mm diam., colony white, then turning purple (similar to OAC555), cracking and turning the media dull orange (lighter than OAC789). **Conidiophores:** *Nodulisporium*-like. **Conidiogenous cells:** Simple, mainly straight or sometimes curled, cylindrical, (5.8−) 21.8 (−28.8) · (2.2−) 2.8 (−3) μm (n = 15), arising directly from hyphae and stretching toward the apex, sometimes dichotomously branched, dimorphic, without collarete, hyaline. Short conidiophores (3−) 4.8 (−5) · 2 μm (n = 15). **Conidia:** macroconidia fusiform, (40.1−) 60.6 (−74.6) · (3−) 3.5 (−5) μm (n = 30), 5–7-septate, slightly curved, hyaline, smooth; microconida cylindrical (11.6−) 16.5 (−25) · (2.2−) 2.8 (−3.6) μm (n = 30), 1–2-septate, hyaline, smooth. **Habitat:** Growing on aged lithographs of Instituto de Investigaciones en Arte (Universidad de Costa Rica). **Notes:** This species, because of the *Nodulisporium*-like conidiophore, is similar to *Coniochaeta ershadii*, especially in the size of the conidiogenous cells. The conidia produced by *C. ershadii* are prominently smaller^33^ than those present in *C. cipronana*; the presence of macro- and microconidia is also a distinguishing character.

The new fungal species described belong to *Periconia* Tode and *Coniochaeta* (Sacc.) Cooke genera (see Supplementary Figures S2 and S3). *Periconia* is a polyphyletic genus Pleosporales (Dothideomycetes, Ascomycota), with a complicated taxonomy and a poorly understood phylogeny^32^. This genus has been widely reported as a common endophyte from the roots of several plants, like a *Periconia* species isolated from *Piper longum* producing metabolites with a high pharmacological potential^34^ and the melanized hyphae are believed to protect the fungi from environmental oxidation^35^. Some species have been reported as parasites in leaves of *Xanthium strumarium* and *Ipomea muricara* in India and others as decomposers in bamboo statches^36^. *Coniochaeta* (Coniochaetaceae, Coniochaetales, Sordariomycetes, Ascomycota) was introduced as a subgenus of *Rosellinia* De Not. for species with hairy perithecia but differing by the absence of amyloid asci in their sexual stages^33^. Many *Coniochaeta* conidiophores produce *Lecytophora*-like structures. Like *Periconia*, *Coniochaeta* requires further taxonomic and phylogenetic studies^37^. About 70 species and six synonyms are included in the genus *Coniochaeta* and most of the isolates are reported from dung, necrotic wood, soil and plant surfaces^38^.

### Cellulase activity of the fungal isolates

Assay of the cellulase activity showed that 95% of the samples produce extracellular enzymes that break down cellulose into smaller oligosaccharides or monosaccharides, as evident from the clear zone observed after staining the plates with Gram’s iodine (see Table 2 and Supplementary Figure S4). This fraction that includes the two new species (sample #19: *Periconia epilithographicola* and sample #21: *Coniochaeta cipronana*) also comprehends species of *Arthrinium, Aspergillus, Chaetomium, Cladosporium, Colletotrichum, Penicillium*, and *Trichoderma*, being the first four commonly reported with cellulolytic activity^31,39–45^. These observations are congruent with the habitat of restricted carbon sources, in which sheets or laminae made of fibers of cellulose pulp were the support material for the growth of microorganisms.

**Table 2:** Enzymatic indices of the isolates on CMC agar stained with Gram Iodine after incubation for seven days.

Isolate#	Enzymatic index
4	No activity
5	3,3 ± 0,2
6	1,91 ± 0,07
7	2,87 ± 0,05
8	0,92 ± 0,02
9	1,6 ± 0,1
10	1,62 ± 0,08
11	2,74 ± 0,02
12	1,47 ± 0,09
13	1,243 ± 0,005
15	2,87 ± 0,03
16	1,47 ± 0,03
17	1,199 ± 0,004
19	1,861 ± 0,002
20	1,17 ± 0,08
21	1,57 ± 0,06
22	1,086 ± 0,006
23	1,39 ± 0,03*
26	0,80 ± 0,06
Control: *P. ostreatus*	1,8 ± 0,1

*EI was measured after 24 h.

Importantly, 32% of the total isolates had a significantly superior enzymatic index relative to a positive control (*P. ostreatus*), i.e., isolates #5 (*Penicillium chrysogenum*), #7 (*Cladosporium tenuissimum*), #11 (*Cladosporium angustisporum*) and #23 (*Trichoderma* cf*. longibrachiatum*). Other studies have characterized these species as effective cellulase producers^16,40^. Isolates #5 (*Penicillium chrysogenum)* and #23 (*Trichoderma* cf. *longibrachiatum*) had an outstanding performance relative to the positive control and the rest of the isolated fungi. Specifically, isolate #5 presented an enzymatic index for cellulose activity almost twice of that of the positive control. Isolates of these species not only have presented important cellulase activity but also have been the object of study for their capacity to produce xylanases^46^, or tanases^47^.

The case of isolate # 23 (*Trichoderma* cf. *longibrachiatum*) was even more striking. For this fungus, EI is reported for 24 h (see Table 2) because after 7 days (the period in which the other isolates were measured) the microorganism had covered the entire Petri plate, evidence of an accelerated growth and a large capacity to use the CMC as the sole source of carbon. The result (1.39 ± 0.03) was slightly smaller than the positive control (1.8 ± 0.1, measured after seven days). However, as previously mentioned, isolate # 23 was measured at 24 h. This result implies a large rate of enzymatic (cellulase) production from fungus #23 in a medium rich in cellulose, relative to the rest of the fungi studied, which is important for the development of biotechnological applications and industry. Many studies have featured this species as a fungus with great cellulase activity^48–51^. Many commercial cellulases can be purchased in purified form after production with this species (e.g. C9748 Sigma-Aldrich or E-CELTR from Megazyme). Investigations with isolation # 23 will continue to evaluate its potential to degrade lignocellulosic residues from agricultural activity in Costa Rica (e.g., wastes from pineapple production).

In summary, in isolating, identifying and characterizing the cellulolytic activity of the fungi responsible for the biodegradation of a nineteenth-century collection, several species of fungi were found to have the ability to produce cellulases. In addition, two new species of fungi were identified and named *Periconia epilithographicola* sp. nov. and *Coniochaeta cipronana* sp. nov., which also have cellulolytic activity. A knowledge of the microorganisms that colonized the Bernard Romain Julien collection belonging to Universidad de Costa Rica will allow the development of strategies directed to the conservation of these ancient lithographs. This work also contributes to the knowledge of new species with cellulolytic activity, which is a topic of perennial interest for biotechnology because of the important role of fungal cellulolytic enzymes in commercial food processing, performing the hydrolysis of cellulose during drying of beans, in the textile industry and laundry detergents, in the conversion of biomass into industrially important solvents or fuels, and their potential application for the bioremediation of wastes.

## Electronic supplementary material

Supplementary information
