# Supplementary material for: Two new cellulolytic fungal species isolated from a 19th-century art collection
Source: Sci Rep. 2018 May 10;8:7492. doi: 10.1038/s41598-018-24934-7 (PMC5945893; doi:10.1038/s41598-018-24934-7)
Supplement: Supplementary file 1 — Supplementary information [file 41598_2018_24934_MOESM1_ESM.docx]

Supplementary Material

**Two new cellulolytic fungal species isolated from a 19^th^-century**

**art collection**

Carolina Coronado-Ruiz^1,2^, Roberto Avendaño^1^, Efraín Escudero-Leyva^2,3^, Geraldine Conejo-Barboza^4,5^, Priscila Chaverri^2,3,6^ & Max Chavarría^1,2,4*^

^1^Centro Nacional de Innovaciones Biotecnológicas (CENIBiot), CeNAT-CONARE, 1174-1200 San José (Costa Rica). ^2^Centro de Investigaciones en Productos Naturales (CIPRONA), Universidad de Costa Rica, 11501-2060 San José (Costa Rica). ^3^Escuela de Biología, Universidad de Costa Rica, 11501-2060 San José (Costa Rica). ^4^Escuela de Química, Universidad de Costa Rica, 11501-2060 San José (Costa Rica). ^5^Instituto de Investigaciones en Arte (II Arte), 11501-2060 San José (Costa Rica). ^6^Department of Plant Science and Landscape Architecture, University of Maryland, College Park, MD 20742 Maryland (USA).

***Correspondence to:** Max Chavarría

Escuela de Química & Centro de Investigaciones en Productos Naturales (CIPRONA)

Universidad de Costa Rica

Sede Central, San Pedro de Montes de Oca

San José, 11501-2060, Costa Rica

Phone (+506) 2511 8520.  Fax (+506) 2253 5020

# E-mail:[max.chavarria@ucr.ac.cr](mailto:max.chavarria@ucr.ac.cr)

**Supplementary table S1**. Accession numbers of sequences deposited in GeneBank.

| **Isolates** | **Identification** | Accesion number  (ITS) | Accesion number (actin) |
| --- | --- | --- | --- |
| 4 | *Cladosporium sphaerospermum* | MF422149 | MF422168 |
| 5 | *Penicillium chrysogenum* | MF422150 | MF422169 |
| 6 | *Penicillium westlingii* | MF422151 | MF422170 |
| 7 | *Cladosporium tenuissimum* | MF422152 | MF422171 |
| 8 | *Aspergillus niger* | MF422153 | MF422172 |
| 9 | *Cladosporium* spp. | MF422154 | -- |
| 10 | *Arthrinium arundinis* | MF422155 | MF422173 |
| 11 | *Cladosporium angustisporum* | MF422156 | MF422174 |
| 12 | *Aspergillus versicolor* | MF422157 | -- |
| 13 | *Chaetomium* cf. *subglobosum* | MF422158 | MF422175 |
| 15 | *Cladosporium angustisporum* | MF422159 | MF422176 |
| 16 | *Cladosporium cladosporioides* | MF422160 | MF422177 |
| 17 | *Chaetomium* cf. *subglobosum* | MF422161 | MF422178 |
| 19 | *Periconia epilithographicola*sp. nov. | MF422162 | MF422179 |
| 20 | *Chaetomium* cf. *subglobosum* | MF422163 | MF422180 |
| 21 | *Coniochaeta cipronasensis* sp. nov | MF422164 | MF422181 |
| 22 | *Aspergillus niger* | MF422165 | MF422182 |
| 23 | *Trichoderma* cf. *longibrachiatum* | MF422166 | MF422183 |
| 26 | *Colletotrichum kahawae* | MF422167 | MF422184 |

## Supplementary Figures


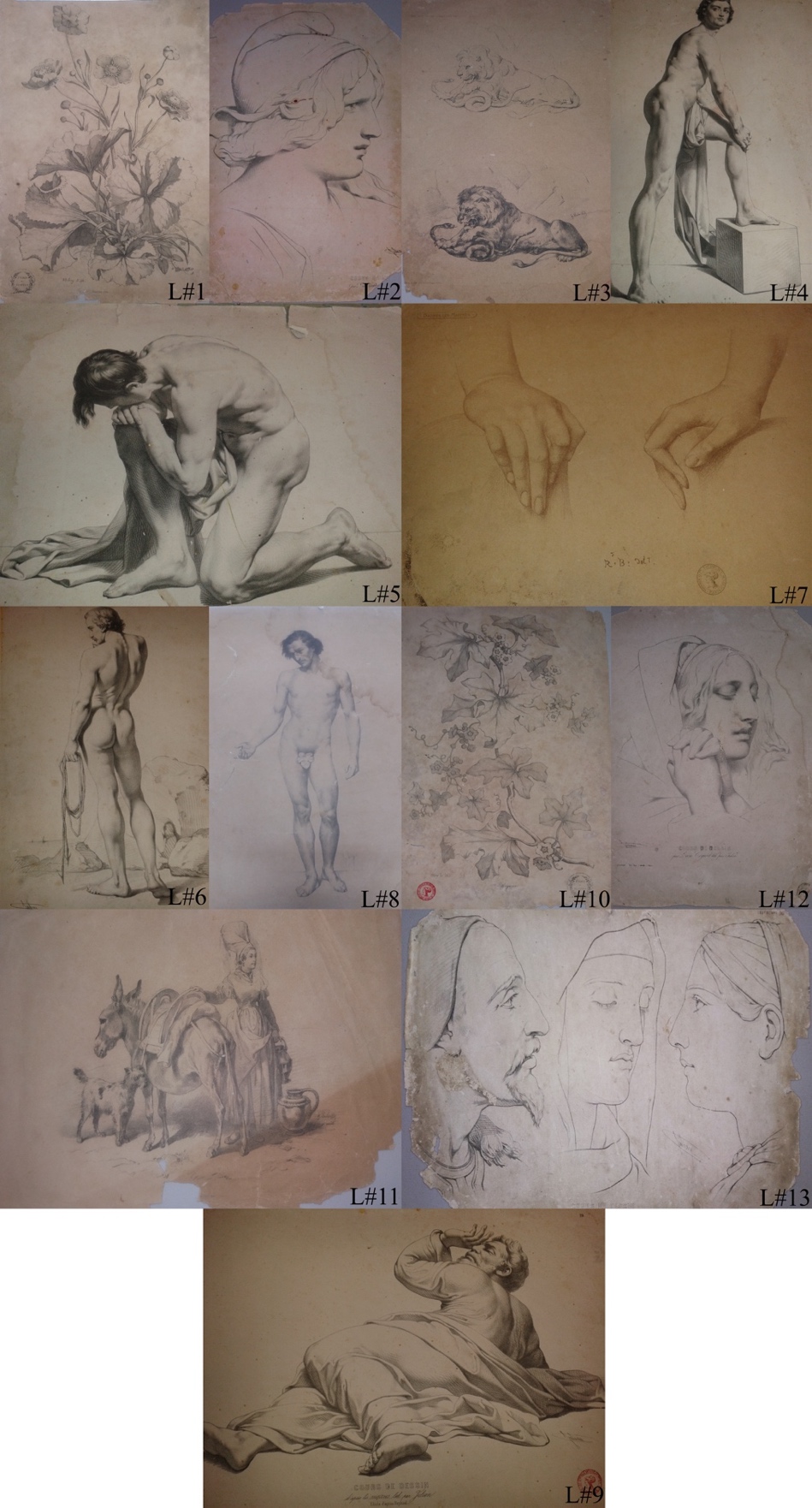


**Supplementary Figure 1.** **Photographs of 13 selected lithographs in which fungi were isolated.** We thank Dr. Salomón Chaves (Instituto de Investigaciones en Arte) for authorizing the use of images from the collection of drawings by Bernard Romain Julien in this manuscript.


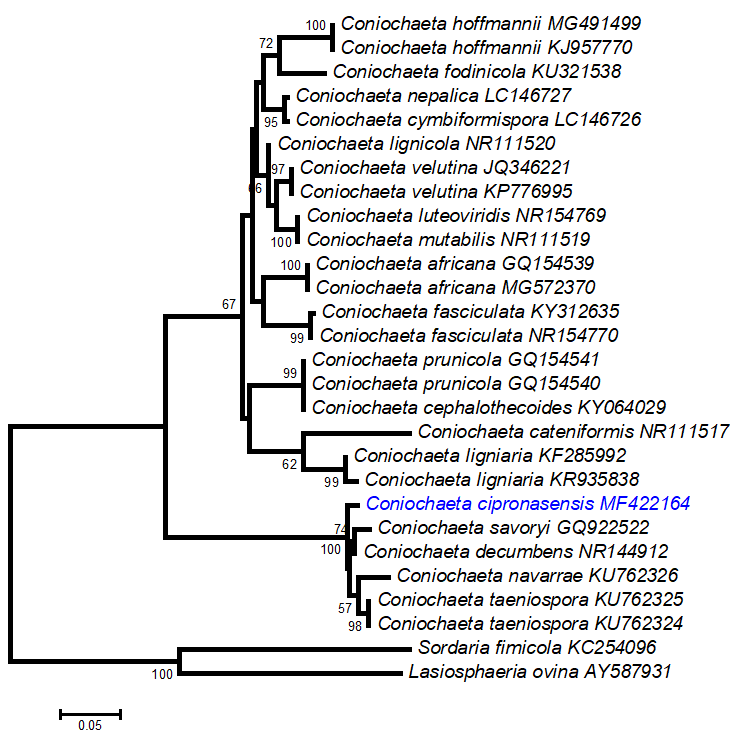


**Supplementary Figure 2.** Maximum-likelihood (ML) tree of *Coniochaeta* based on the ITS region. ML bootstrap proportion greater than 50% is presented at the nodes (in total 1000 bootstrap replications were calculated). *Coniochaeta cipronasensis* sp. nov. in blue. The tree was rooted to *Sordaria fimicola* and *Lasiosphaeria ovina* in the Sordariomycetidae.


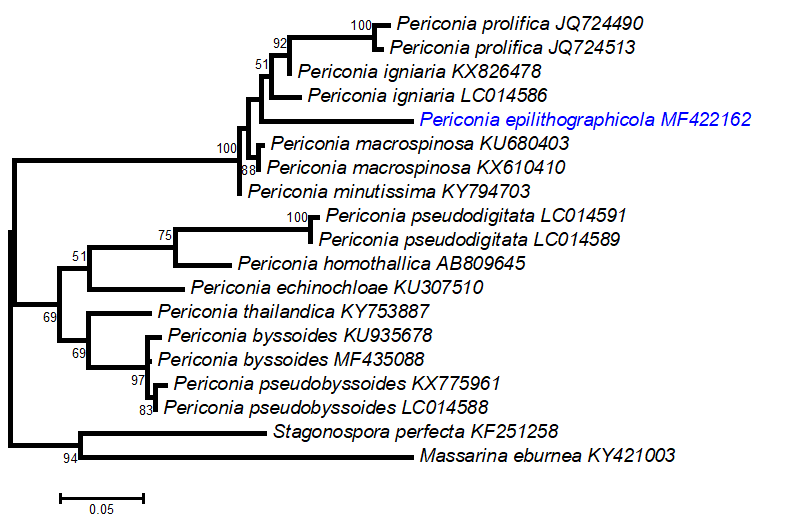


**Supplementary Figure 3.** Maximum-likelihood (ML) tree of *Periconia* based on the ITS region. ML bootstrap proportion greater than 50% is presented at the nodes (in total 1000 bootstrap replications were calculated). *Periconia epilithographicola* sp. nov. in blue. The tree was rooted to *Stagonospora perfecta* and *Massarina eburnea* in the Massarinaceae.

**
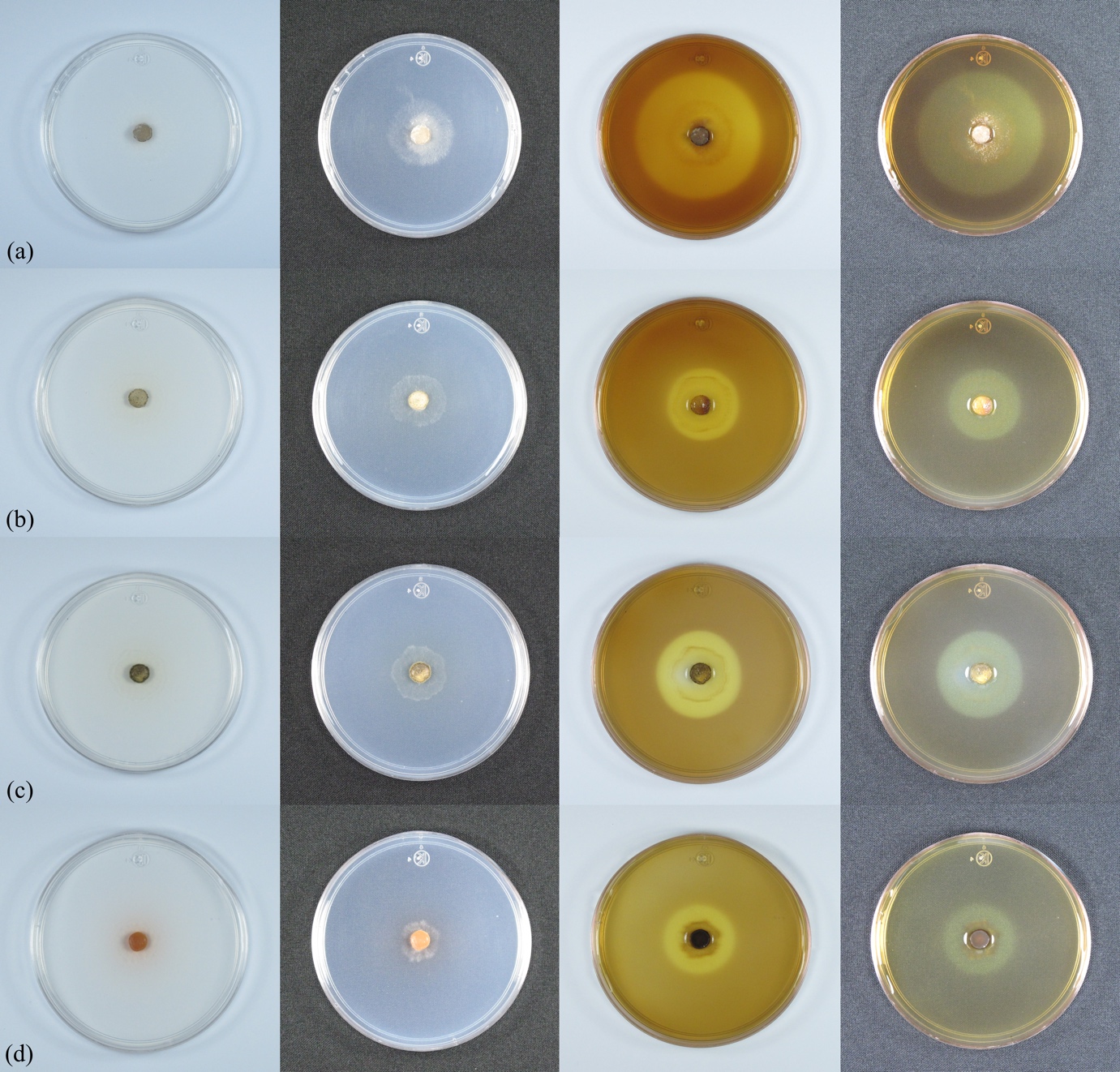
**

**Supplementary Figure 4. Qualitative determination of cellulolytic activity.**Columns 1 and 2 show carboxymethyl cellulose (CMC) plates of selected isolates before treatment with Gram's iodine dye. Cultures are shown in white (column 1) and black (column 2) backgrounds to improve contrast and facilitate its visualization. Columns 3 and 4 show the plates after the treatment with Gram's iodine dye also in white (column 3) and black (column 4) backgrounds. Each row corresponds to (a) *Arthrinium arundinis, (b) Chaetomium*cf.*subglobosum, (c) Chaetomium cf. subglobosum and (d) Coniochaeta cipronasensis* sp. nov.
